# How Does Postural Control in Patients with Functional Motor Disorders Adapt to Multitasking‐Based Immersive Virtual Reality?

**DOI:** 10.1002/mdc3.13961

**Published:** 2024-01-04

**Authors:** Marialuisa Gandolfi, Angela Sandri, Zoe Menaspà, Laura Avanzino, Elisa Pelosin, Christian Geroin, Denis Vidale, Mirta Fiorio, Michele Tinazzi

**Affiliations:** ^1^ Department of Neurosciences, Biomedicine and Movement Sciences University of Verona Verona Italy; ^2^ Neuromotor and Cognitive Rehabilitation Research Centre (CRRNC) University of Verona Verona Italy; ^3^ Neurorehabilitation Unit AOUI Verona Italy; ^4^ IRCCS Ospedale Policlinico San Martino Genoa Italy; ^5^ Department of Experimental Medicine Section of Human Physiology, University of Genoa Genoa Italy; ^6^ Department of Surgery, Dentistry, Pediatrics and Gynecology University of Verona Italy; ^7^ Khymeia SRL Padua Italy

**Keywords:** balance, attention, functional motor disorders, exergaming, virtual reality

## Abstract

**Background:**

Motor symptoms in functional motor disorders (FMDs) refer to involuntary, but learned, altered movement patterns associated with aberrant self‐focus, sense of agency, and belief/expectations. These conditions commonly lead to impaired posture control, raising the likelihood of falls and disability. Utilizing visual and cognitive tasks to manipulate attentional focus, virtual reality (VR) integrated with posturography is a promising tool for exploring postural control disorders.

**Objectives:**

To investigate whether postural control can be adapted by manipulating attentional focus in a 3D immersive VR environment.

**Methods:**

We compared postural parameters in 17 FMDs patients and 19 age‐matched healthy controls over a single session under four increasingly more complex and attention‐demanding conditions: simple fixation task (1) in the real room and (2) in 3D VR room‐like condition; complex fixation task in a 3D VR city‐like condition (3) avoiding distractors and (4) counting them. Dual‐task effect (DTE) measured the relative change in performance induced by the different attention‐demanding conditions on postural parameters.

**Results:**

Patients reduced sway area and mediolateral center of pressure displacement velocity DTE compared to controls (all, *P* < 0.049), but only under condition 4. They also showed a significant reduction in the sway area DTE under condition 4 compared to condition 3 (*P* = 0.025).

**Conclusions:**

This study provides novel preliminary evidence for the value of a 3D immersive VR environment combined with different attention‐demanding conditions in adapting postural control in patients with FMDs. As supported by quantitative and objective posturographic measures, our findings may inform interventions to explore FMDs pathophysiology.

Functional motor disorders (FMDs) are disabling neurological conditions at the intersection of neurology and psychiatry.^1^ Part of functional neurological disorders (FNDs), they are clinically evaluated as abnormal movements caused by impaired brain networks that manifest distressing motor, sensory, and/or cognitive symptoms.^2^ The incidence ranges from 4 to 12 per 100,000 population per year, accounting for 15–20% of patients seeking neurological care,^3^, ^4^, ^5^ where positive signs prove incongruent with organic movement disorders.^3^, ^6^, ^7^ People with FMDs (PwFMD) often report gait and balance disorders (along with dystonia, weakness, and tremor), which increase the risk of falls and disability.^3^, ^8^, ^9^, ^10^ Like other movement disorders, FMDs are associated with long‐term disability, poor quality of life, distress, and an economic burden on health and social care.^11^, ^12^ Since the early 2000s, breakthroughs in PwFMD pathophysiology and management^1^, ^13^, ^14^, ^15^ have helped set the disorder into a biopsychosocial framework, where predisposing, precipitating, and perpetuating factors lead to symptoms’ manifestation.^16^ FMDs are involuntary but learnt altered movement patterns caused by abnormal self‐directed attention and movement prediction, resulting in a movement generated without a normal sense of agency.^1^, ^3^, ^17^, ^18^, ^19^ Within this perspective, multidisciplinary intensive rehabilitation, supported by telemedicine, is a widely recognized strategy for the PwFMD management.^20^, ^21^, ^22^, ^23^ Retraining movement through diverted attention and changing maladaptive symptoms‐related behaviors can reduce disability and improve patients’ quality of life.^20^, ^21^, ^22^, ^23^ Future research directions require developing interventions for treating specific functional symptoms based on the pathophysiological features of FMDs, such as the altered focus of attention, sense of agency, and belief/expectations.^1^, ^3^, ^15^, ^23^, ^24^, ^25^ Some techniques for specific symptoms to normalize movement have been recommended, mainly focusing on weakness, gait disturbances, tremors, and dystonia.^23^ No specific interventions for postural control disturbances have been identified so far. Within this context, an innovative approach to tackle and manage postural deficits would involve accurately manipulating perceptual information and subsequent attentional control over movements, targeting the fundamental pathophysiological features of FMDs.^1^, ^3^, ^6^, ^15^, ^17^, ^25^, ^26^ This can be achieved through virtual reality (VR) technology, which offers a promising avenue to optimize motor learning in a safe, challenging, and motivating environment, stimulating sensorimotor and cognitive processes simultaneously.^27^, ^28^ The use of immersive VR (ie, through a Head‐Mounted display, VR‐HMD) is particularly relevant because it allows interaction with the virtual environment under visually manipulated conditions.^29^, ^30^, ^31^, ^32^, ^33^, ^34^, ^35^ Exploring VR‐based changes in postural control by combining VR‐HMD with posturographic measurements is of great interest in specific neurological diseases (eg, Parkinson's disease, stroke, multiple sclerosis)^29^, ^30^, ^31^, ^32^, ^33^, ^34^, ^35^ under the assumption that attentional manipulation could shape postural strategies.^36^, ^37^

Indeed, the immersive VR environment can be programmed with specific characteristics (ie, first or third perspective, type of scenery, type of environment) and multisensory feedback (ie, visual and/or auditory) suited to explore different levels of attentional conditions in interaction with multiple tasks in a controlled environment.^26^, ^38^ Despite the great potential of VR in improving postural control in patients with movement disorders,^33^, ^39^ its application in PwFMD has been very limited, with no evidence up‐to‐now on its effects on functional postural disorders.^19^, ^40^, ^41^ The possible justification for using VR technology in PwFMD over what is currently used are the following: (1) the immersive VR environment can be programmed with specific characteristics (i.e., type of scenery, type of environment) and multisensory feedback (ie, visual and/or auditory) suited to explore different levels of attentional conditions in interaction with multiple tasks in a controlled environment;^26^, ^38^ (2) VR‐based strategies in PwFMD can lowered the ineffective (presumed deliberated) “higher‐level” control of posture by introducing progressive attentional demanding conditions.^6^, ^42^, ^43^, ^44^ This effect hints at the role of attentional mechanisms in functional improvement, unlike other neurological diseases such as Parkinson's and multiple sclerosis, in which motor performance worsens.^45^, ^46^

Before conducting a full‐scale study to demonstrate the effectiveness of immersive VR rehabilitation protocols in PwFMD through a randomized controlled trial (NCT05581134), we performed an exploratory hypothesis‐generating study by investigating the effects of an immersive VR environment on postural control in PwFMD compared to healthy controls (HC). The protocol called for an increasing attentional demand spectrum of conditions to verify the effects on posture control. We hypothesized that the application of immersive VR associated with progressive attention‐demanding conditions would promote more effective use of postural control strategies in PwFMD as measured by posturography. It would be implemented clinically as part of the quantitative assessment of functional postural control disturbances to support the FMDs diagnosis.^6^

## Methods

### Study Design

For this observational, exploratory cross‐sectional study, 17 PwFMD with a clinically definite diagnosis^47^ (mean age, 45.25 ± 15.20 years, 76.47% women) and 19 HC (mean age, 41.58 ± 16.58 years, 73.8% women) were enrolled from the Parkinson's Disease and Movement Disorders Unit of the AOUI (Verona, Italy).

### Participants

Inclusion criteria were age 18 years or older, lower limb functional motor symptoms and/or sensory nature, and normal or corrected to normal vision. Exclusion criteria were history of epilepsy, prominent dissociative seizures, need for assistive devices to maintain upright posture, other comorbidities that could interfere with postural control (dizziness, vestibular disorders, orthopedic or cardiovascular comorbidities), and Mini‐Mental State Examination score <24/30. Duration and severity of functional motor symptoms were measured with the objective‐rated Simplified Functional Movement Disorders Rating Scale (S‐FMDRS, range, 0–54; higher scores indicate worse rating).^48^ All participants gave their written, informed consent to participate. The study was carried out following the tenets of the Helsinki Declaration and approved by the local Ethics Committee (Prog. 3571CESC – JP‐VR‐19).

### Posture Assessment

**Figure 1:**
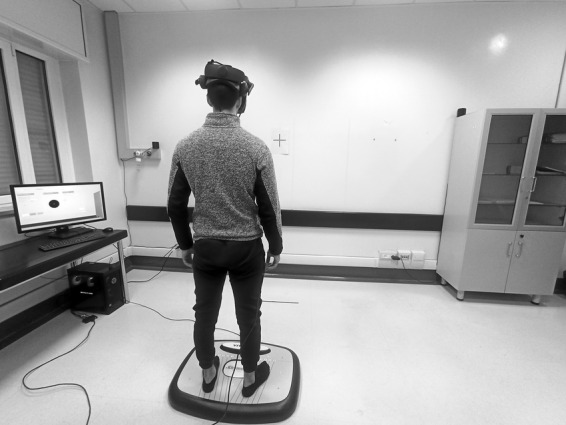
Posture assessment. The subjects stood on an electronic monoaxial platform with arms held alongside the body, and the foot position was standardized. For Condition 1, they did not wear a VR headset. For Conditions 2, 3, and 4, they wore it.

The experimental setup is illustrated in Fig. 1. Subjects were always asked to maintain standing on a posturographic platform with arms held alongside the body and eyes open while focusing on a visual target (red cross, visual target distance 1.5 m).^6^, ^49^ Displacement of the pressure center (CoP) was recorded with an electronic monoaxial platform (Khymeia, Italy) during four consecutive increasingly more complex and attention‐demanding conditions (180 s in duration each), described below. As a proxy for postural control, we measured the sway area (mm^2^), the length of the CoP trajectory (mm), and the mean velocity of CoP displacement in the anteroposterior (AP) and the mediolateral (ML) (mm/s) direction.^6^

The sway area corresponds to the surface of the ellipse, covering 95% of the computed trajectory of the CoP, and indicates the amplitude of body sway.^50^ The length of the CoP trajectory is the overall distance covered by the successive positions of the moving CoP. The AP and ML CoP displacement means velocity is the normalized version of the length of the CoP, according to task duration, on the x‐axis and the y‐axis, respectively.^50^ The former indicates the distal‐proximal movement strategy (ankle strategy) and, thus, control of ankle extensor activity through anticipatory strategies. The latter indicates the proximal‐distal movement strategy to maintain the CoP within the base of support (hip strategy). Higher posturographic measurements suggest less ability to maintain balance in static conditions and a significantly higher risk of falling.^50^

### Virtual Reality Setting

Two custom‐made 3D VR environments were displayed on a virtual reality headset (Vive Pro Eye, HTC Corporation) with stereoscopic stimulus rendered on a Nvidia GeForce GTX 1060 graphics card, a resolution of 1440 × 1600 pixels per eye, and a diagonal field of view of 110 degrees.^29^, ^51^

The simplest scenario consisted of a 3D room‐like condition in which the subject found themselves in the photo‐realistic virtual copy of the real room where the experiment was held.^48^ The more complex VR environment was represented by a 3D city‐like scene (Khymeia, Italy) in which the subject found themselves at a street corner in an urban‐like setting, surrounded by buildings, trees, traffic lights (fixed elements), and pedestrians (moving elements) acting as distractors. The pedestrians were depicted in high‐contrast‐colored shirts (yellow, red, blue), walked at three different speeds, and changed in number during the task. A background sound effect was also present (traffic noise). In both environments, subjects found a visual target (fixation cross) projected at 1.5 m from themselves.

### Study Protocol

**Figure 2:**
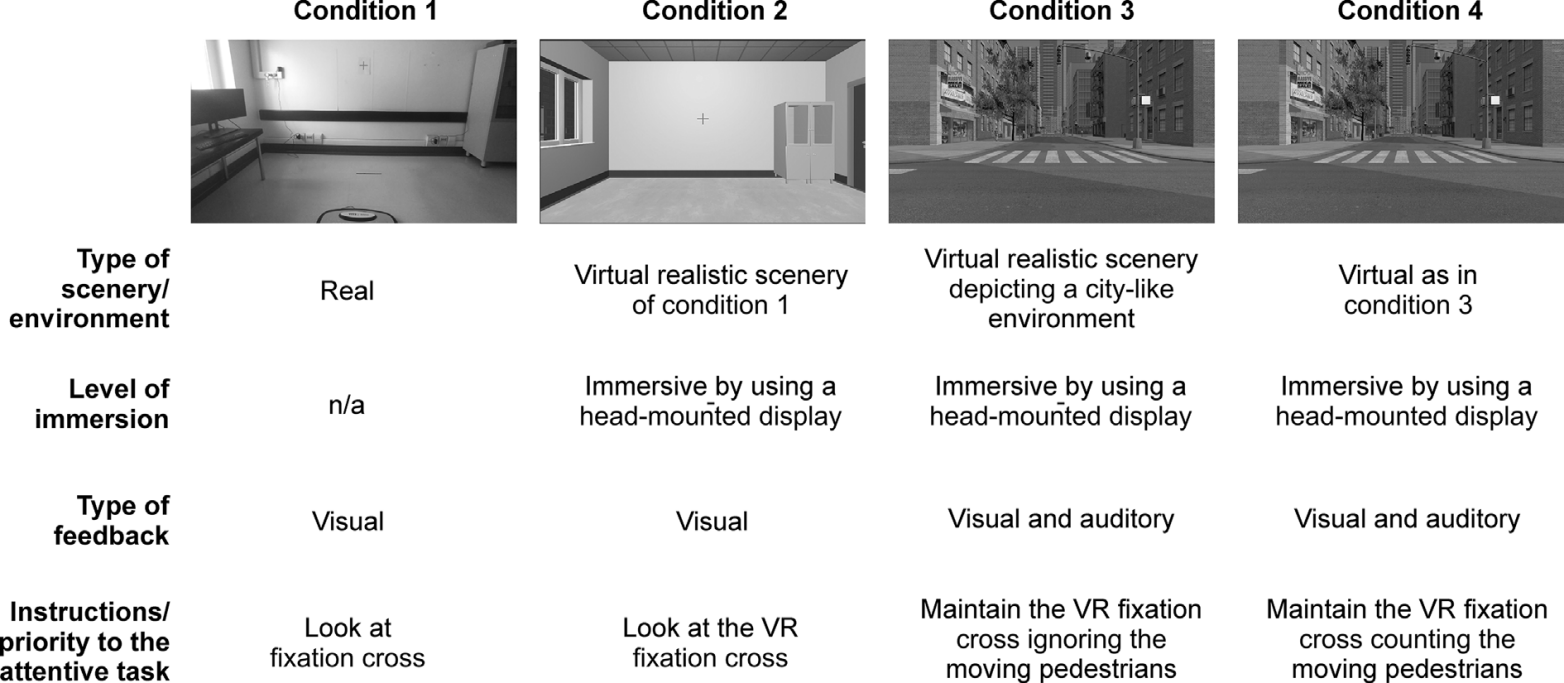
Virtual reality features and study protocol. Condition 1 refers to the real environment. Conditions 2, 3, and 4 refer to custom‐made 3D VR environments, including the more demanding tasks. n.a., not applicable; VR, Virtual reality.

The study protocol comprised four consequential, increasingly complex, and attention‐demanding conditions (Fig. 2).^6^ Condition 1 was the simplest attentional condition in which the patient underwent posturographic assessment in the real environment focusing on the fixation cross. This allowed observing postural control under a simple visual task in the real environment. Condition 2 consisted of maintaining the standing position while immersed in the 3D room‐like environment that replicated real‐world scenarios. Using the 3D room‐like environment, we investigate whether the visual task in the simpler VR environment could elicit similar postural control responses as the real environment.

In Conditions 3 and 4, the patient underwent posturographic assessment while immersed in the most complex 3D city‐like scene. Two types of attention‐demanding tasks were required in this setting. In Condition 3, the patient had to maintain fixation while ignoring distractors (the moving elements). This task involves more complex attentional mechanisms than the previous one, requiring stronger inhibitory visual control. It has been described in the literature as a fixation dual‐task. In condition 4, participants had to maintain the fixation while counting distractors. Therefore, they performed an attentional and a cognitive task simultaneously (mental tracking task).^52^ This condition was the most demanding because, other than maintaining fixation, it requires holding information in the mind while performing the cognitive process (fixation‐cognitive dual task).

The VR environment was synchronized with the stabilimeter recording. The clinical and instrumental assessment was done on the same day. Before starting the VR conditions, subjects could explore the VR environment for about 10 s to familiarize themselves. The eye‐tracking system monitored the fixation point. Trials in which the subject was not maintaining eye fixation on the cross were removed. Comments on the VR experience through qualitative information collection and side effects due to VR exposure that might have occurred during the experiment (ie, motion sickness, dizziness, nausea) were assessed for each subject by the experimenter at the end of the procedure.

### Statistical Analysis

Descriptive statistics included frequency tables for categorical variables and mean and standard deviation or median and interquartile ranges for continuous variables. Fisher's exact test was used to check for between‐group sex differences. Non‐parametric tests were applied because the data were not normally distributed (Shapiro–Wilk test, *P* < 0.05).

As a preliminary analysis, we compared posturographic raw data recorded in conditions 1 and 2 to ensure condition 2 as the reference condition for subsequent analyses (simplest VR visual task).

The Dual‐Task Effect (DTE) expressed in percentage (%) was computed for each posturographic parameter and subject to evaluate the effects on performance induced by the more complex and attention‐demanding conditions (conditions 3 and 4) to the simpler and less demanding (condition 2) according to the following formula.^53^

$$\;\mathrm{DTE}\;\left(\%\right)=\frac{\;\text{Dual task performace}-\text{Single task performance}}{\text{Single task performance}}\times 100$$

Here, the single task represents the performance in condition 2, where the subject had to perform the simpler task. The dual‐task refers to the performance in Conditions 3 and 4, where the subject had to perform the motor task (maintaining postural control) and, concurrently, the increasingly demanding additional attentional tasks (visual inhibition and mental tracking tasks). Since higher postural excursions indicate postural instability,^54^ a higher DTE (>0) reflects worse postural control induced by the dual task to the single task. In contrast, a lower DTE (<0) indicates better postural control induced by the dual task to the single task (less postural instability). As this was an exploratory study, we did not correct the analyses for multiple comparisons. The Wilcoxon test for independent samples was used separately for between‐group comparisons in each condition. The Wilcoxon test for related samples was used for within‐group comparisons. As supplementary analyses, we compared the raw posturographic data among the VR‐based conditions (conditions 2, 3, and 4) through the Friedman and Wilcoxon tests for related samples for within‐group comparisons. The alpha level was 0.05. All analyses were performed with RStudio 2022.07.1 Software statistics (© 2009‐2022 RStudio, PBC).

## Results

### Study Sample Characteristics

Table 1 presents the clinical and demographic characteristics of the FMDs and the HC. All patients complained of imbalance symptoms. There were no differences in age and sex between the two groups (all, *P* > 0.39).

**TABLE 1:** Clinical and demographic characteristics of the study sample

	PwFMD (*n* = 17)	HC (*n* = 19)	Test & *P*‐value
Mean age, years (SD)	45.25 (15.20)	41.58 (12.00)	*W* = 126; *P* = 0.40
Women, no. (%)	13 (76.47)	14 (73.78)	OR = 0.87; *P* = 1
Mean duration symptoms, years (±SD)	3.77 (5.25)		
Clinical characteristics – no. (%)			
Motor symptoms			
Tremor	10 (58.82)		
Weakness	12 (70.59)		
Dystonia	4 (23.53)		
Tics	2 (11.76)		
Parkinsonism	3 (17.65)		
Gait impairments	12 (70.59)		
S‐FMDRS (SD)			
Total score (0–54)	12.35 (6.67)		

Abbreviations: HC, healthy controls; OR, odds ratio; PwFMD, people with functional motor disorders; SD, standard deviation; S‐FMDRS, simplified functional movement disorders rating scale; %, percentage; W, Wilcoxon rank sum test.

### Preliminary Analyses

Figure S1 presents the mean and the standard error of posturographic raw data in conditions 1 and 2. FMDs showed significantly higher posturographic parameters (worse performance) in both conditions on all measures compared to HC (all, *P* < 0.029), except for the ML CoP displacement mean velocity in condition 1 (*W* = 102, *P* = 0.061). No significant within‐group differences were noted for FMDs on any measure (all, *P* > 0.48). In HC, there was a significant increase in the AP CoP displacement mean velocity (*V* = 42, *P* = 0.032) and a decrease in the ML CoP displacement mean velocity (*V* = 151, *P* = 0.023) in condition 2 compared to 1.

### Dual Task Effect of VR‐Based Tasks on Posturographic Parameters

**Figure 3:**
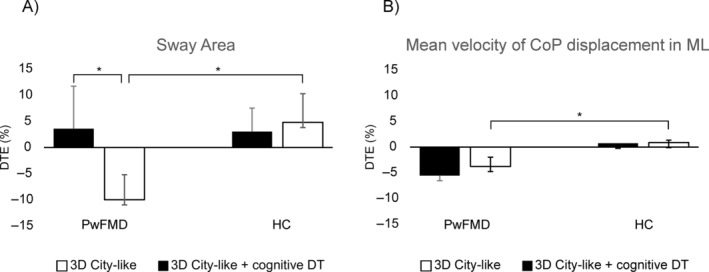
Dual‐task effect in FMDs and healthy controls. Dual‐task effect. Dual Task Effect (DTE) was calculated for each subject for sway area (A) and velocity of CoP displacement in the ML direction (B), reflecting changes in performance induced by Condition 3 (in gray) or 4 (in light blue) concerning Condition 2; Data were analyzed using non‐parametric tests; *P < 0.05. *, indicates that the corresponding median values were significantly different (*P* < 0.05) in the measures of sway area for PwFMD on Condition 4 compared to Condition 3 and to the HC. In addition, changes were significantly different in the measures of ML displacement for the patients on Condition 4 compared to the HC.

All subjects have provided positive feedback regarding their experience during the VR conditions. No side effects were recorded in the patients or the controls. The accuracy in distractors detection on Condition 4 was 95%. Compared to controls, FMDs significantly decreased their DTE for sway area in Condition 4 (*W* = 212; *P* = 0.048) but not in Condition 3 (*W* = 177; *P* = 0.64), indicating lower sway area (better postural control) in patients with FMDs while performing the VR fixation‐cognitive dual‐task (Fig. 3A). Similarly, FMDs significantly decreased the DTE for the ML CoP displacement mean velocity on Condition 4 (*W* = 220; *P* = 0.024) but not on Condition 3 (*W* = 223; *P* = 0.052) compared to HC, meaning lower mean velocity of the ML displacement (better postural control) while performing the VR fixation‐cognitive dual‐task condition than the fixation dual task (Fig. 3B). In FMDs, the DTE on the sway area was significantly decreased from Condition 4 to Condition 3 (*V* = 111; *P* = 0.025), meaning a lower sway area (better postural control) during the VR fixation‐cognitive dual‐task with respect to VR fixation dual‐task condition. No other significant differences were found (all, *P* > 0.11). Mean and standard error for the DTE in each group can be seen in Table 2.

**TABLE 2:** Mean (and standard error of the mean) for posturographic parameters and DTE effect in PwFMD and healthy controls

			Raw data			DTE (%)
	Group	Condition 2	Condition 3	Condition 4	Condition 3 versus 2	Condition 4 versus 2
		Mean (SE)	Mean (SE)	Mean (SE)	Mean (SE)	Mean (SE)
Sway area (mm^2^)	PwFMD	113.32† (20.57)	106.04† (14.94)	92.34 (12.93)	3.59 (8.11)	−9.97†* (4.77)
	HC	58.55 (3.63)	59.82 (4.09)	60.54 (4.72)	3.04 (4.48)	4.80 (5.46)
Length of CoP (mm)	PwFMD	2568.6† (152.47)	2433.3† (99.72)	2450.79† (137.22)	−3.65 (2.10)	−3.40 (1.92)
	HC	2071.18 (69.65)	2076.14 (65.20)	2084.97 (68.01)	0.42 (0.65)	0.77 (0.60)
Vel ML (mm/s)	PwFMD	32.79† (2.29)	30.11 (1.34)	31.04 (2.11)	−5.55 (2.82)	−3.78* (1.81)
	HC	26.32 (1.08)	26.47 (1.03)	26.54 (1.07)	0.74 (0.53)	0.88 (0.47)
Vel AP (mm/s)	PwFMD	24.34† (1.25)	23.90† (1.13)	23.20† (1.04)	−1.26 (1.57)	−3.07† (2.10)
	HC	19.53 (0.51)	19.42 (0.47)	19.50 (0.47)	−0.35 (1.11)	−0.05 (1.09)

*Note*: Raw data refers to posturographic parameters measured during each condition. The Dual‐Task Effect reflecting changes in performance induced by the dual task (Conditions 3 or 4) with respect to the single task (Condition 2) was calculated for each subject for each posturographic parameter. Data were analyzed using non‐parametric tests. Friedman test for raw data (Conditions 2, 3, and 4) was not significantly different in both PwFMD and HC groups; † indicates significant between‐group differences on the same condition (*P* < 0.05) by the Wilcoxon rank sum exact test for independent samples; *, indicates significant within‐group differences between different condition (*P* < 0.05) by the Wilcoxon signed‐rank test for related samples for the Dual Task Effect.

Abbreviations: DT, dual‐task; HC, healthy controls; PwFMD denotes the Functional Motor Disorder group; SE, Standard Error; Vel AP, mean velocity of CoP displacement in the anteroposterior direction; Vel ML, mean velocity of CoP displacement in the mediolateral direction; %, percentage.

### Supplementary Analyses

Table 2 presents the mean and the standard error of posturographic raw data of Conditions 2, 3, and 4. FMDs reported higher posturographic raw parameters compared to HC (worse performance) on all conditions (all, *P* < 0.03), except for the ML CoP displacement mean velocity on Conditions 3 and 4 (all, *P* > 0.68). No significant within‐group comparisons were found for Conditions 2, 3, and 4 in FMDs and HC (all, *P* > 0.87).

## Discussion

Using quantitative and objective posturographic measures combined with a 3D immersive VR environment, this exploratory study is the first to investigate whether postural control can be adapted to increasingly more complex and attention‐demanding conditions in PwFMD. The main finding is that postural control was positively shaped only in the immersive VR city‐like environment combined with the fixation‐cognitive task, proved by the significant reduction in the DTE for the sway area and the mean velocity of CoP displacement in the mediolateral direction in the patients compared to healthy controls.

The rationale for designing a higher demanding cognitive task (condition 4) in a VR environment was related to the need to induce an external focus of attention, which has been shown to improve posturographic measures on a complex cognitive task.^55^, ^56^ We may hypothesize that the same mechanism occurred in our experiment, assuming that the most demanding cognitive task in a VR environment could have engaged participants in a highly distracting task that might have sustained their attention for a longer time compared to a real environment. The novelty of our study is the use of an immersive VR 3D environment, developed by taking into account the current knowledge on FMDs (pathophysiology and management) and the use of an experimental step‐by‐step protocol to explore the possible effects of VR in PwFMD combined with an instrumental (objective) assessment of performance. No side effects were reported after a single VR session, and all subjects provided positive feedback on the VR experience. A feasibility study shares this observation with weekly VR sessions lasting 5–20 min for FND (upper limb tremor) that reported no adverse events.^41^ Devising strategies to improve motor symptoms and postural control disturbances in PwFMD is central to reducing disability while performing daily‐life activities. Indeed, VR technology potentially transfers trained movement patterns to daily life functional activities by replicating real‐life scenarios,^57^ enhancing the ecological validity of the results.^51^, ^58^ The current technology also enables easy applicability of such tools in clinical practice, considering their user‐friendly nature. However, concerns about the costs of acquiring certified medical device technologies and developing specific software must be considered.

### Differences in Posturographic Parameters between Real and 3D Room‐Like Conditions

Consistent with the exploratory nature of this study, we found that VR alone without any additional task, like in the 3D room‐like condition, did not affect postural control in PwFMD. This finding differs from the postural changes observed in the HC in this condition, as revealed by the increase in the velocity of CoP displacement in the AP direction and the decrease in the ML direction. These changes are consistent with the HC's ankle strategy to adjust to the new environment and probably re‐weight the sensory information depending on sensory context,^59^, ^60^ that is, increase normal distal‐proximal activation (ankle strategy). In the 3D room‐like condition, subjects wore VR‐head‐mounted goggles, and therefore, this finding suggests that simply wearing the headset could change postural stability in young, healthy controls.^49^ Our findings partially agree with those by Imaizumi et al (2020), who noted in healthy individuals, a CoP displacement increase on both AP and ML axes and in the CoP displacement velocity in the AP direction and sway area.^49^ A plausible explanation for the inconsistency between our findings and those by Imaizumi et al (2020) is that in our protocol, the same visual (real or virtual) fixation point (red cross) was present in the real room and in the 3D room‐like conditions to promote visual stabilization of postural control.^49^

In contrast, Imaizumi (2020) used a natural and virtual scene of a clean, smooth white wall surface, where subjects could explore the environment by moving only their eyes, thus having less visual input to help stabilize postural control.^49^ The lack of change in postural control in PwFMD ought not to be interpreted as a positive effect of VR because their posturographic performance was significantly worse in the 3D room‐like conditions compared to the HC. This suggests that visual cues from the 3D room‐like environment did not provide sufficient external reference to influence attentional and visual postural control mechanisms. Indeed, the virtual room environment was the same in dimension, color, and furnishings as in the real room.

### Effect of 3D VR‐Based Tasks on Posturographic Parameters

The significant reduction of the dual‐task effect on sway area and the velocity of CoP displacement in the ML direction suggests that the 3D city‐like virtual context combined with a cognitive dual task may provide sufficient elements to improve postural control in PwFMD patients compared to HC. This was not the case with the same 3D city‐like virtual context without concurrent cognitive tasks.

A possible explanation is that the 3D city‐like virtual environment combined with the cognitive tasks further increased external attentional focus with a consequent decrease in sway area and velocity of CoP displacement in the ML direction compared to the VR environment without the cognitive task. The reason why external focus and cognitive tasks might improve postural control is thought to result from a more automatic type of postural control.^56^ Richer et al (2017) described this effect in which posturographic measures were found to improve when subjects externally focused on a more complex cognitive task compared to baseline and internal focus.^56^ We may assume that in our experiment, condition 4 served not only as a more distal point of focus from the body but also sustained attention longer than the simpler external focus task as in the fixation task (Condition 3), leading to a further increase in stability and less conscious interference with postural control.^55^ By directing conscious attention away from sway on the cognitive task, the external focus may have enabled automatic processes to control sway more efficiently in the patients than in the controls.^61^

Previous studies have advised caution with VR usage in PwFMD^40^ because motor tasks with manipulated feedback alone might not be sufficient to alter patients’ performance, also given the contradictory results in cognitive dual‐tasking performed in the real environment.^21^, ^22^, ^23^ Accordingly, we found significant results only when we combined the motor task (maintaining postural control) with the manipulated feedback (VR) and the cognitive task. In brief, having more complex distracting tasks may help improve the condition of HC^56^ and PwFMD.^41^ We may speculate that such exposure was more efficient in the patients than in the controls because it acted directly on one of the pathophysiological mechanisms underlying FMDs, that is, abnormal allocation of attention, and so might have influenced other altered mechanisms as well (sense of agency and belief/expectations), whereas simple dual‐tasking not combined with VR might not be so comprehensive.^21^, ^22^, ^23^ However, it should be acknowledged that these effects might be transient, possibly influenced by the novelty of the VR environment. An electrophysiological study provided evidence for slow sensory information processing in patients with FNDs, suggesting a reduced attention allocation to objective body signals,^62^ which could explain the shift towards an overemphasized feedforward signal.

Ours is the first study to explore VR technology in PwFMD with balance disorders. Indeed, customized tools are more effective than non‐specific systems such as commercial exergames.^26^, ^33^ We included a healthy control group to collect normative data and quantitative postural assessment to assess changes in performance objectively. Overall, our preliminary results justify the use of VR technology as it offers a unique and innovative approach to explore postural control disturbances, with the potential to uncover new insights on diagnosis and therapeutic strategies. We cannot conclude that VR, combined with increasingly more complex and attention‐demanding conditions, can be used as an intervention per se in managing PwFMD.

Nonetheless, it can provide a valuable tool in the long‐term management of these patients, as recommended for other movement disorders,^33^ and prepares the way for a randomized controlled trial to test the effectiveness of VR as a rehabilitation tool. The main limitations of the present study are the lack of validated neuropsychological tests to assess attentional deficits and subjective measurements to document the mismatch between symptom perception and objective postural assessment and the lack of a control condition where the cognitive task is performed in a non‐immersive environment. These findings could be strengthened by conducting a two‐phase study hypothesis‐testing/replicating by running only those tasks and tests that reached significance in our first set of experiments.

## Author Roles

(1) Research Project: A. Conception, B. Organization, C. Execution; (2) Statistical Analysis: A. Design, B. Execution, C. Review and Critique; (3) Manuscript Preparation: A. Writing of the First Draft, B. Review and Critique.

M.T.: 1A, 2C, 3B

M.F.: 1A, 2C, 3B

M.G.:1B, 1C, 2A, 2B, 3A

A.S.: 2A, 2B, 2C, 3A

Z.M.: 1B, 1C

C.G.: 1B, 3B

L.A.: 1B, 3B

E.P.: 1B, 3B

D.V.: 1C, 3B

## Disclosures

**Ethical Compliance Statement:** Name of the institutional review board or ethics committee that approved the study: Comitato etico per la Sperimentazione Clinica (CESC) delle Province di Verona e Rovigo, AOUI Verona (Italy)–Prog. 3571CESC–JP‐VR‐19. Declaration of patient consent: all participants gave their written, informed consent to participate. Affirmation that all authors have read and complied with the Journal's Ethical Publication Guidelines: we confirm that we have read the Journal's position on issues involved in ethical publication and affirm that this work is consistent with those guidelines.

**Funding Sources and Conflicts of Interest:** Funding was received for this work from the University of Verona ‐ Joint Project 2019 (JP‐VR‐19), Italy. The authors declare no conflicts of interest relevant to this work.

**Financial Disclosures for Previous 12 Months:** The authors declare no additional disclosures to report.

## Supporting information


**Figure S1.** Mean (and standard error of the mean) for posturographic parameters in patients and healthy controls on conditions 1 and 2. PwFMD denotes the FMDs group; HC, healthy controls; Vel ML, mean velocity of CoP displacement in the mediolateral direction; Vel AP, mean velocity of CoP displacement in the anteroposterior direction. Data were analyzed using non‐parametric tests. *, indicates significant differences (*P* < 0.05). Patients had significantly worse posturographic parameters than controls, except for Vel ML at condition 1. Controls reduced their Vel ML and increased their Vel AP at Condition 2.
