# Supplementary figures and images for: How Does Postural Control in Patients with Functional Motor Disorders Adapt to Multitasking‐Based Immersive Virtual Reality?
Source: Mov Disord Clin Pract. 2024 Jan 4;11(4):337–45. doi: 10.1002/mdc3.13961 (PMC10982601; doi:10.1002/mdc3.13961)

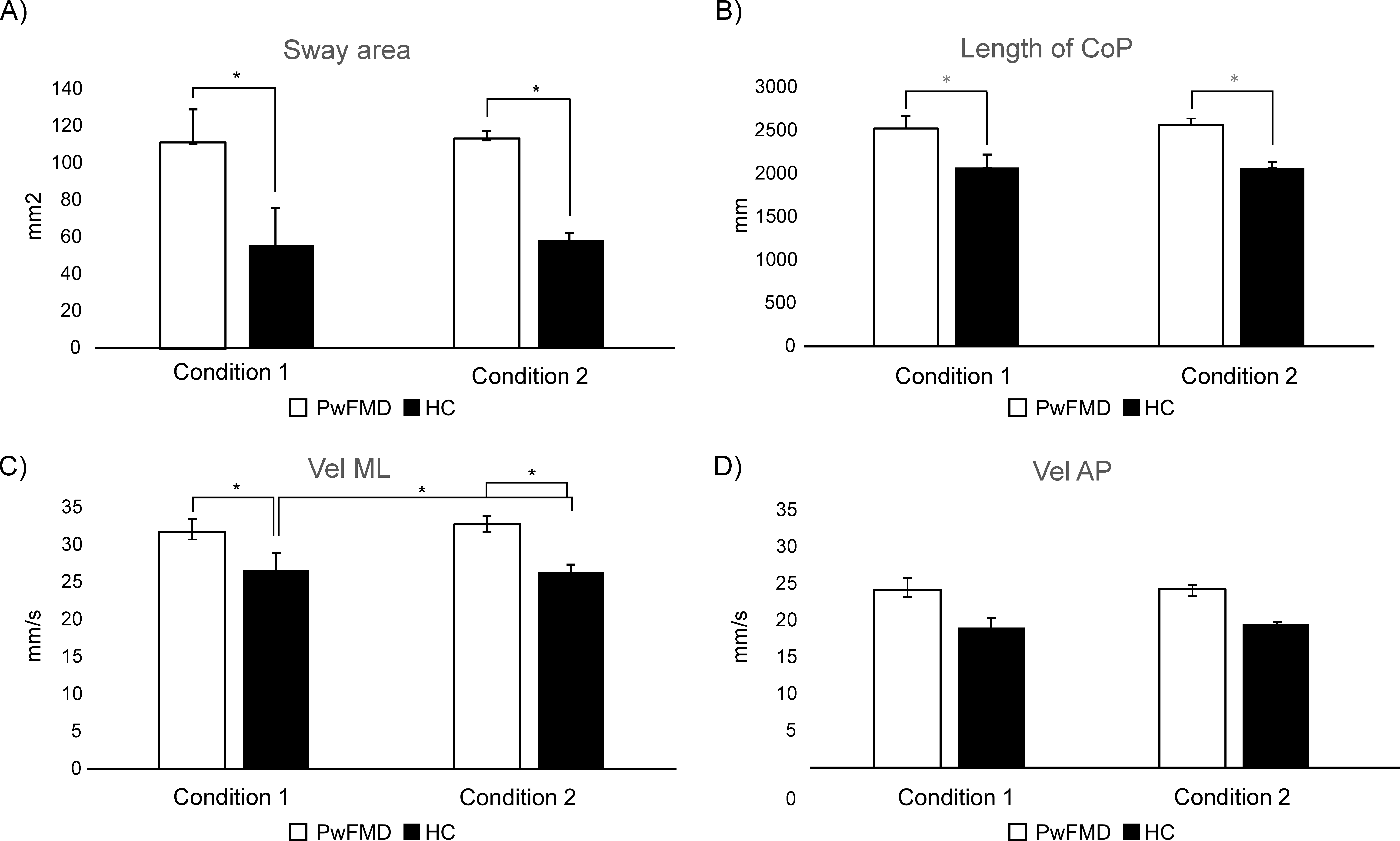

Supplement: Supplementary file 1 — Figure S1. Mean (and standard error of the mean) for posturographic parameters in patients and healthy controls on conditions 1 and 2. PwFMD denotes the FMDs group; HC, healthy controls; Vel ML, mean velocity of CoP displacement in the mediolateral direction; Vel AP, mean velocity of CoP displacement in the anteroposterior direction. Data were analyzed using non‐parametric tests. *, indicates significant differences (P < 0.05). Patients had significantly worse posturographic parameters than controls, except for Vel ML at condition 1. Controls reduced their Vel ML and increased their Vel AP at Condition 2. [file MDC3-11-337-s001.tif]
